# Surgical technique for biological fixation of closed segmental tibial fractures by the Less Invasive Stabilization System (LISS)

**DOI:** 10.1051/sicotj/2018046

**Published:** 2018-11-14

**Authors:** Osama Gamal, Ahmed Shams

**Affiliations:** Orthopaedic Department, Faculty of Medicine, Menoufia University Shebin Elkom, Menoufia Egypt

**Keywords:** LISS, Segmental, Tibia fracture.

## Abstract

*Introduction*: This prospective case series study aimed to assess the value of the Less Invasive Stabilization System (LISS) to treat closed Segmental Tibial Fractures (STFs) using a proposed surgical technique.

*Materials and methods*: Between August 2010 and January 2014, 21 consecutive recently (within 1 week) closed STFs that matched the inclusion criteria were enrolled. Patients were treated with the 13-hole LISS plate. All patients were followed up every 2 weeks for the first 2 months, then every month for the rest of the first 6 months and then every 6 months thereafter. Patients were assessed radiologically during the follow-up appointments and clinically at the final visit by the Lower Extremity Functional Scale (LEFS) to evaluate the result.

*Results*: The mean time to union of the proximal fracture was 15.72 ± 2.78 (range: 12–20) weeks and for the distal fracture was 20 ± 2.22 (range: 16–24) weeks, excluding delayed union in three patients. All patients except the three showed radiological observable callus in a mean duration of 4.95 (range: 3–7) weeks. The mean final follow-up LEFS was 72.4 (range: 60–80).

*Conclusion*: The mean time to union of the proximal fracture was shorter than the distal fracture. The use of LISS to treat closed STFs using the proposed surgical technique has proved to give favorable results. Further studies using the described technique are needed to justify the achieved results.

*Level of evidence*: IV (Prospective case series).

## Introduction

Segmental tibia fractures (STF; AO 42-C2) are defined by two or more distinct fracture lines separating an interposed cortical segment(s) excluding butterfly fragmentation [1]. The middle fragment can vary between 3 and 20 cm and comminution can be present at both fracture sites [2]. They are often caused by high-energy and some by low-energy traumas as sports and twisting. STFs are often part of multiple injuries; nearly 50% of them are open and account for 6.5–8% of tibia fractures [3,4]. Unfortunately, little attention is paid to STFs despite the plethora of literature on tibia fracture [3].

Management of these fractures is challenging and carries a high complication rate. The currently preferred modality is intramedullary nail fixation. Needless to say that reduction is often problematic because of the unstable intercalary segment that may need open placement of a bone clamp to hold and stabilize it during reaming and may also need extra plating and blocking screws [5]. Other favorite treatment methods include external fixators with multiple different frames and versatility and nonoperative treatment using plaster of Paris or functional braces [6].

Recently, with the advances in plate osteosynthesis and evolving fixed angle locking screws and the sub-muscular plating concept, biological fracture fixation with the purpose of preserving tissue vascularity around the fracture site has become possible.

In this prospective case series study, a question was proposed if the Less Invasive Stabilization System (LISS) could be favorably used to treat closed segmental tibia fractures (STF) by the proposed surgical technique.

## Materials and methods

**Figure 1:**
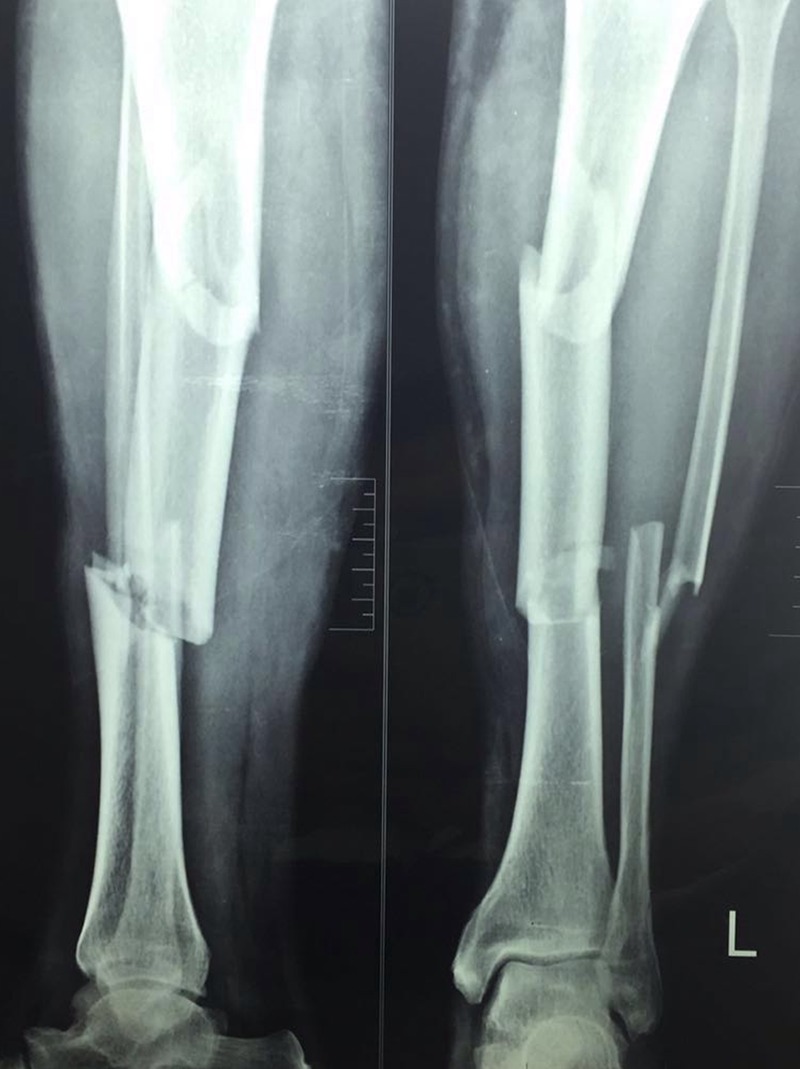
AP and Lat radiographs showing SFT; AO 42-C2.

Between August 2010 and January 2014, 21 consecutive recently (within 1 week) closed STFs (Figure 1) were treated with the 13-hole LISS plate (Synthes USA, Paoli, PA).

There were 12 males and 9 females with a mean age of 41.2 (range: 19–67) years. Nine patients had right-side injury, while 12 had left-side injury. All fractures resulted from high-energy traumas.

Those patients were treated at an academically supervised level A trauma center by two consultant surgeons after local research ethics committee approval. The study treatment choice was discussed with the patients and consents were taken. Inclusion criteria were patients who are skeletally mature (age ≥18 years) with displaced closed segmental tibial fractures (each fracture is not involved into the rule of the square). The exclusion criteria included OTA types (42-C2.3, 42-C3.1), skeletally immature (age ≤18 years), open fracture or bone loss, infected blisters, proved compartment syndrome, an ipsilateral lower limb fracture, pathological fractures, peripheral neurologic or vascular damage, uncontrolled diabetes mellitus, refusal to participate and loss during follow-up.

Plain radiographic evaluation of the fractures was done by anteroposterior (AP) and lateral (Lat) views of the whole tibia including the knee and ankle joints.

All patients were operated on in the supine position on a radiolucent table allowing complete lower leg imaging under C-arm guidance. A bump was placed under the patient's buttock to direct the patella anteriorly to ease true AP and Lat images. A tourniquet was used in all patients and suitable padding under the uninvolved extremity was placed and secured in place.

**Figure 2:**
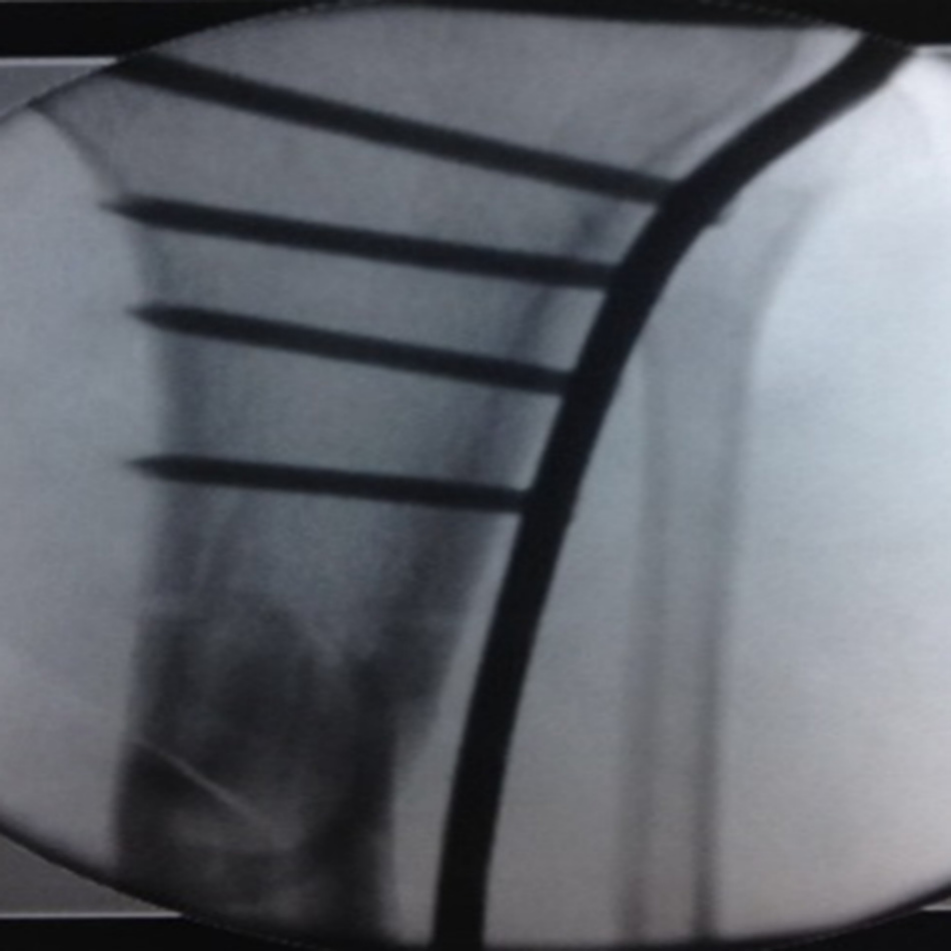
Proximal end of the plate fixed to the proximal tibia in a proper position.

A lateral curved (hockey stick) incision was used, starting at the Gerdy's tubercle and extending about 5 cm in a distal direction. The proximal tibia LISS insertion guide (left or right) main unit and radiolucent extension were assembled. The Tibialis anterior muscle was dissected starting about 5 mm from the tibial ridge and retracted about 3 cm distally. The plate was inserted from proximal to distal between the muscle and the periosteum under fluoroscopic control with its distal end kept in constant contact with the tibia. To find the proximal plate placement against the lateral tibial plateau, the plate was moved distally and then proximally. Tactile feedback pointed to correct plate location on the lateral plateau flare. For the LISS plate, a 2.0 mm Kirschner (K) wire was inserted parallel to the plateau to provide initial fixation. Proper location of the plate proximal end was confirmed with the C-arm guided Lat view to corroborate that it was centered on the proximal tibia and afterward the diaphysis locking screws were positioned through the intramedullary canal center (Figure 2).

**Figure 3:**
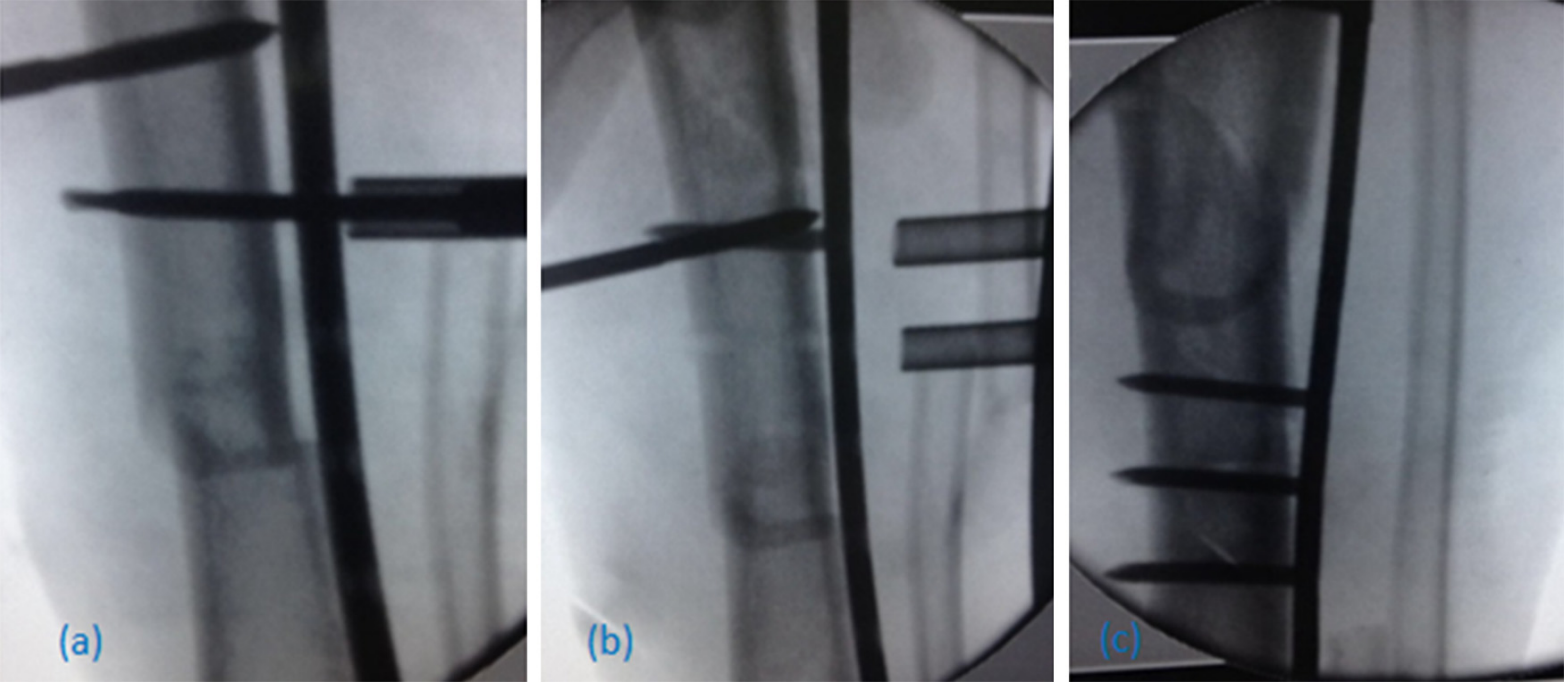
Intercalary segment fixation: (a) Reduction using Schanz pin and the whirlybird. (b) First screw in place. (c) Final screws fixation.

The intercalary segment was adjusted about the proximal and distal segments for both alignment and rotation, using a threaded 4.5 mm Schanz pin inserted medially in the midsegment for manipulating it back to its position. This Schanz pin facilitated nearly a full control over this floating segment to align it back to both proximal and distal fracture sites. All these were aided by closed external clamping to pull the midsegment as close as possible to the plate and by using the whirlybird device provided in the LISS instrumentation system; this segment was further approximated to the plate (Figure 3a). No attempt was made to open the fracture site and a minimum of two locking screws, inserted through percutaneous stabs aided by C-arm guidance, were used to fix this segment (Figure 3b and c).

**Figure 4:**
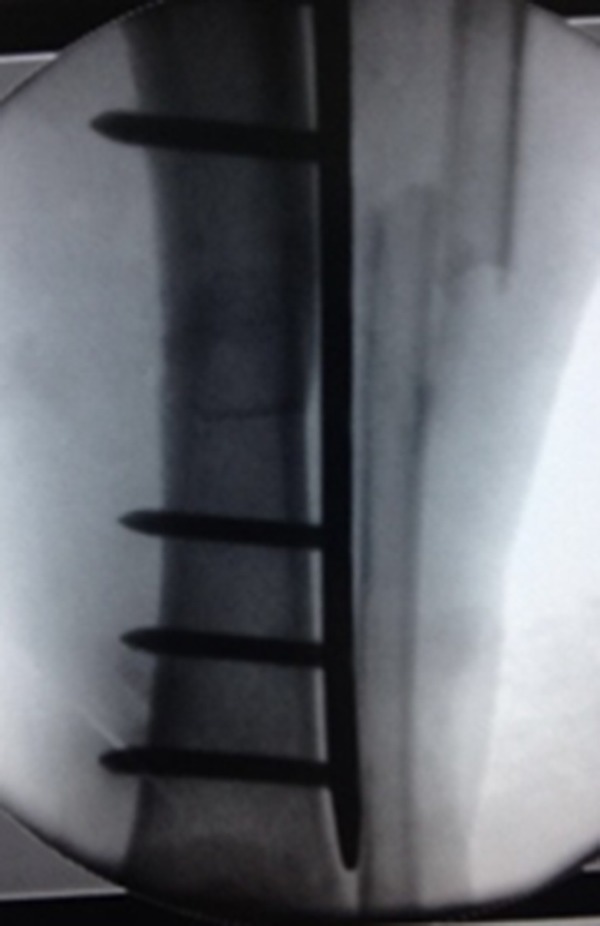
Distal end of the plate fixed to the distal fragment.

For the distal fragment, once the plate had been inserted and positioned properly, with reduction reconfirmed, a slightly extended distal incision was made and ended at the last holes of the plate (No. 13) and marked using the insertion sleeve. This was done to recheck the plate position and see the superficial peroneal nerve to avoid its damage. This fragment was adjusted about the intercalary segment for both alignment and rotation (Figure 4).

During every step of the instrumentation technique, the foot position is rechecked to prevent malrotation and a minimum of three (5 mm titanium locking self-drilling) screws were inserted in each main fracture fragment. Finally, the proximal and distal incisions were sutured after tourniquet release and hemostasis.

Above knee, posterior plaster splint was applied with leg elevation over a pillow to decrease postoperative edema with anti-edema medications. Active bleeding was monitored. Active toe movement was started after recovery from anesthesia. Radiographs of the whole tibia including the knee and ankle joints in both AP and Lat views were checked. Intravenous antibiotics were started and continued for 24 h postoperatively, switched to oral antibiotics on the second postoperative day and continued for 1 week. Analgesics were given on need.

**Figure 5:**
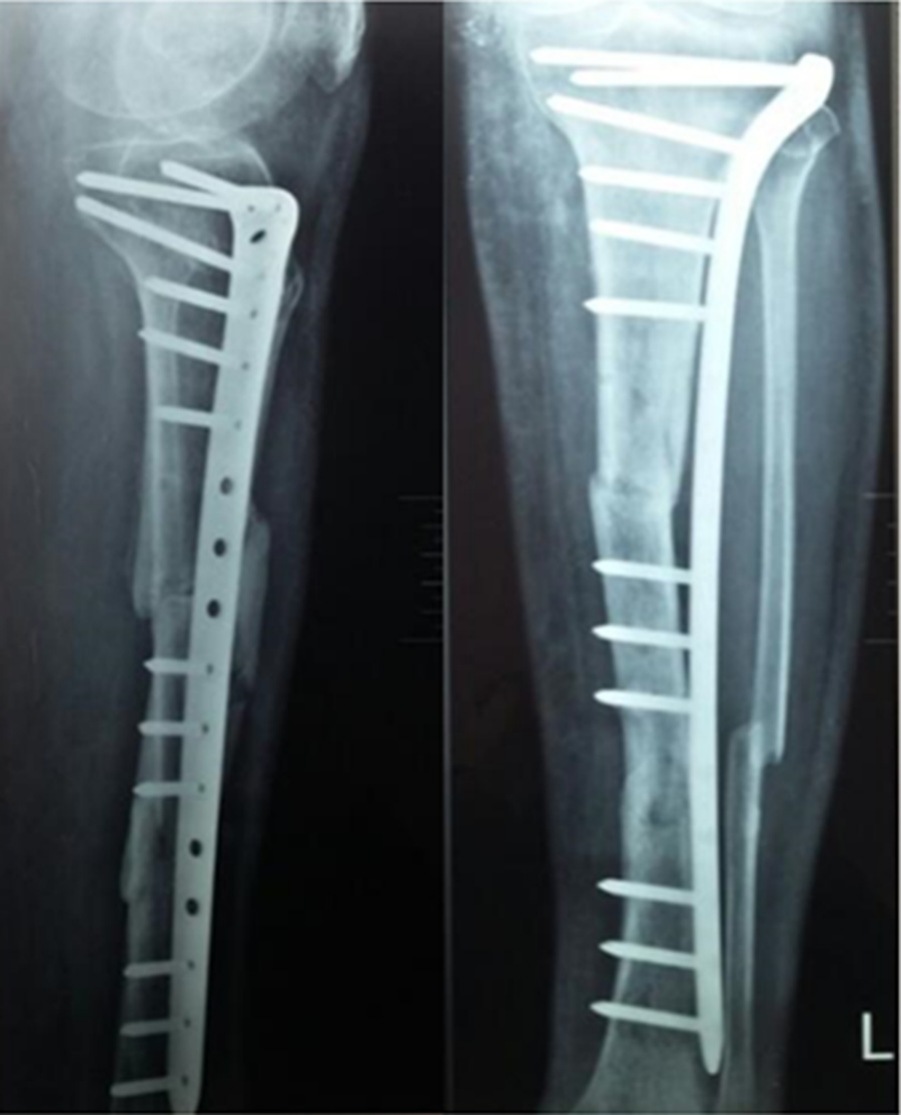
AP and Lat radiographs showing healed proximal and distal fractures 20 weeks postoperatively.

Patients were followed up every 2 weeks for the first 2 months, then monthly for the rest of first 6 months and then every 6 months thereafter. Two weeks postoperatively, the sutures and splint were removed and a hinged knee brace was used by all patients. About 6–8 weeks postoperatively, a follow-up AP and Lat radiographs were made, and if callus was obvious, partial weight bearing was started. About 12–16 weeks postoperatively, follow-up radiographs were made, and if complete union was obvious on radiographs, full weight bearing was started. Patients were allowed full weight bearing when there was no pain at the fracture site with radiological signs of bone union (Figure 5) using the Radiographic Union Score for Tibial fractures (RUST) [7]. Limb rotation and alignment were evaluated at all follow-up visits. The minimal follow-up was 24 months. At the last follow-up visit, the Lower Extremity Functional Scale (LEFS) was used to assess the functional outcome by a surgeon who was unaware of the patients' information [8].

### Statistical analysis

For statistical analysis, the paired *t*-test was used. Normality assumption of data distribution was tested by Shapiro–Wilk test. The *p*-value was set at 0.05 so that *p*-values >0.05 are statistically nonsignificant (ns), *p*-values ≤0.05 are significant (s) and *p*-values ≤0.01 are highly significant (hs).

## Results

Table 1 shows patient characteristics. The mean time to union (Figure 5) of the proximal fracture was 15.72 ± 2.78 (range: 12–20) weeks and for the distal fracture was 20 ± 2.22 (range: 16–24) weeks, excluding delayed union in three patients. This difference between the two means was highly significant (*P* < 0.001) (95% CI = –4.27778 ± 1.2742). All patients except the three showed radiologically observable callus in a mean duration of 4.95 (range: 3–7) weeks. Those three patients (14.3%) progressed to delayed union at the distal fracture lines 6 months postoperatively. One of them was treated by endomedullary bone marrow injection and the other two were treated using shock wave stimulation after which healing was uneventful in 8 weeks. Full weight bearing was allowed after observable complete bifocal radiological union. This was after a mean of 18.47 (range: 16–24) weeks. The mean final follow-up LEFS was 72.4 (range: 60–80).

**Table 1:** Patients characteristics.

Characteristic	
Age (years)	41.2 (range: 19–67)
Male:female	12 (57.1%): 9 (42.9%)
Right:left	9 (42.9%): 12 (57.1%)
Trauma (RTA: fall from height)	19 (90.5%): 2 (9.5%)
Injury to surgery (days)	1.95 (range: 0–7)
Follow-up (months)	30 (range: 24–36)

The mean hospital stay was 4.57 (2–12) days. Eleven patients had a history of chronic illnesses (52.4%). No clinically relevant malrotation was noted. No patients had postoperative compartment syndrome. No patients were complicated with superficial peroneal nerve palsy, extensor halluces longus palsy, deep venous thrombosis or pulmonary embolism. There was superficial infection in only one patient, which responded to systemic antibiotic therapy and local wound care. Three patients had implant-related complaints (14.3%) that needed its removal following bone healing, because of the prominent proximal part. There was one patient with 1.9 cm shortening that was detected during initial plating and intentionally allowed because of severe bone comminution around the fracture site.

## Discussion

The treatment key of tibial fractures is to preserve their soft tissue envelope, reduce fracture site interventions and reproduce anatomical length, alignment and rotation while optimizing the union chances. STFs challenge these aforementioned principles and the ability to provide stability with the most standard implants. Not only are the endomedullary blood vessels broken up on two levels but also the intercalary bone segment itself is often separated from the surrounding soft tissues (skin, periosteum and muscle attachments). The resultant precarious blood supply of this segment increases the risk of nonunion, delayed union, infection and added procedures [1]. Boutin was the first to define the poor prognosis of these fractures [9].

In 1976, Langàrd and Bo treated 23 STFs with plate osteosynthesis and found complication rates of 26 and 57% for closed and open fractures, respectively [4]. Also in 1989, in a series of 22 patients treated with plate osteosynthesis, Rommens et al. found a 60% complication rate with greater than 25% chance of wound complications and infection. Not surprisingly, nearly 20% of those tibias went on to develop pseudarthrosis with some progressing to implant failure [10].

As a better understanding of fracture-healing biology has developed, the use of plate osteosynthesis decreased. In most of the reported studies on this subject, the fixation technique was varied, with a prevalence of locked intramedullary nailing (IMN) [11]. Shifting to nailing, the main advantage was their ability to preserve the osseous blood flow by reducing the adjacent tissues disruption [12]. On the other hand, this modality needs surgeon's experience and skills because of the challenging surgical procedures and the more susceptibility to malalignment [13,14]. In addition, these fractures with intermediate segment precarious blood supply are at possibility of secondary injury because of rotation when reaming [10].

Recent studies have achieved favorable results using the minimally invasive plate osteosynthesis (MIPO) as an alternative technique to replace IMN [15,16]. The LISS, after the fragment reposition, is ideal to preserve periosteal, intramedullary vascularization and reduce soft tissue injuries by its sub-muscular insertion compared to conventional open reduction and compression, which is essential for fracture healing. Besides, instead of IMN insertion, LISS does not damage the intramedullary blood supply by preserving its environment.

In the current study, the surgical technique carried out for reduction of the intercalary segment employed a medially inserted threaded 4.5 mm Schanz pin facilitating nearly a full control over this floating segment and manipulating it back to its position aided by closed external clamping and the whirlybird device to approximate this segment to the plate. No attempt was made to open the fracture site, thus preserving the fracture hematoma and reducing the soft tissue envelope injuries.

In this study, the mean time to union of the proximal fracture was 15.72 ± 2.78 (range: 12–20) weeks, and for the distal fracture it was 20 ± 2.22 (range: 16–24) weeks. These results went with those of other studies targeting the same fractures [13,16] and were even shorter than Giannoudis study with the mean time to union of the proximal fracture of 38.8 weeks (range: 10–78 weeks) and 41.4 weeks (range: 12–65 weeks) for the distal fracture [1].

The distal fracture is the common site for delayed union and nonunion, the proposed clarifications for this being the direct injury to the soft tissues covering it and the natural tendency to slow union in this location [17]. In this study, three patients (14.3%) had delayed union at the distal fracture lines 6 months after surgery. One of them was treated by endomedullary bone marrow injection and the other two were treated using shock wave stimulation. The three patients healed in 8 weeks duration.

Three patients had implant-related complaints (14.3%) that needed to be removed following bone healing, because of the prominent proximal part. In Reynders study, seven patients (30.3%) needed LISS plate removal after bone healing for the same reason [16].

There was one patient with 1.9 cm shortening that was noted during initial plate stabilization, and was intentionally allowed because of severe bone comminution at the fracture site. In Reynders study [16], there were two patients of shortening: one by 2.4 cm and the other by 3.6 cm. In the study of Huang et al. [13], one case of shortening about 1.5 cm due to dynamization after 8 weeks.

In this study, no clinically relevant malrotation was noted. No patients had postoperative compartment syndrome. This goes with the study of Huang et al. [13]. No patients were complicated with extensor halluces longus palsy, deep venous thrombosis or pulmonary embolism. No superficial peroneal nerve palsy was noted as a slightly extended distal incision was made, marked using the insertion sleeve in the last hole 13 to see the nerve and avoid its damage as recommended by Reynders [16]. There was superficial infection in only one patient, which responded to systemic antibiotic therapy and local wound care. This result was better compared to related studies of Huang et al. of two deep infections [13] and Reynders of one deep infection [16].

Although this series was rather small because of the relative rarity of these injuries, it was observed that there is a tendency toward better healing with this method, possibly due to the improved elasticity of the bone–plate complex without any high grade of instability of the intercalary bone segment and this goes with the results of another study [18].

This study has some limitations such as the small sample size, lack of comparison group and short follow-up period. More prospective, multicenter, randomized studies with a larger sample size and longer follow-up periods are warranted in the future to further justify the results of the used technique.

In conclusion, the mean time to union of the proximal fracture was shorter than the distal fracture. The use of LISS to treat closed STFs using the proposed surgical technique has proved to give favorable results.

## Conflict of interest

The authors declare that they have no conflict of interest in relation to this article.
